# Supplementary material for: Diverging Effects of Adolescent Ethanol Exposure on Tripartite Synaptic Development across Prefrontal Cortex Subregions
Source: Cells. 2022 Oct 2;11(19):3111. doi: 10.3390/cells11193111 (PMC9561972; doi:10.3390/cells11193111)
Supplement: Supplementary file 1 [file cells-11-03111-s001.zip › cells-1905156-supplementary-final.pdf]

Figure 2A.

Table Analyzed	10th Dose Astrocyte Volume				
Two-way ANOVA	Ordinary				
Alpha	0.05				
Source of Variation	% of total variation	P value	P value summary	Significant?	
Interaction	0.1112	0.8458	ns	No	
Subregion	34.72	<0.0001	****	Yes	
Treatment	0.1706	0.2639	ns	No	
ANOVA table	SS (Type III)	DF	MS	F (DFn, DFd)	P value
Interaction	77421304	3	25807101	F (3, 476) = 0.2718	P=0.8458
Subregion	24169310491	3	8056436830	F (3, 476) = 84.85	P<0.0001
Treatment	118786953	1	118786953	F (1, 476) = 1.251	P=0.2639
Residual	45197895063	476	94953561		
**Tukey's multiple comparisons tes**	**Predicted (LS) mean diff.**	**95.00% CI of diff.**	**Below threshold?**	**Summary**	**Adjusted P Value**
ACC:H2O vs. ACC:EtOH	650.6	-5283 to 6584	No	ns	>0.9999
ACC:H2O vs. mPFC:H2O	-4635	-9828 to 557.9	No	ns	0.1199
ACC:H2O vs. mPFC:EtOH	-2702	-7895 to 2491	No	ns	0.7597
ACC:H2O vs. LO-OFC:H2O	11034	5100 to 16967	Yes	****	<0.0001
ACC:H2O vs. LO-OFC:EtOH	12662	6729 to 18596	Yes	****	<0.0001
ACC:H2O vs. VO-OFC:H2O	11796	5802 to 17791	Yes	****	<0.0001
ACC:H2O vs. VO-OFC:EtOH	11705	5710 to 17700	Yes	****	<0.0001
ACC:EtOH vs. mPFC:H2O	-5285	-10478 to -92.63	Yes	*	0.0427
ACC:EtOH vs. mPFC:EtOH	-3353	-8545 to 1840	No	ns	0.5064
ACC:EtOH vs. LO-OFC:H2O	10383	4450 to 16316	Yes	****	<0.0001
ACC:EtOH vs. LO-OFC:EtOH	12012	6079 to 17945	Yes	****	<0.0001
ACC:EtOH vs. VO-OFC:H2O	11146	5151 to 17141	Yes	****	<0.0001
ACC:EtOH vs. VO-OFC:EtOH	11054	5059 to 17049	Yes	****	<0.0001
mPFC:H2O vs. mPFC:EtOH	1933	-2394 to 6260	No	ns	0.8748
mPFC:H2O vs. LO-OFC:H2O	15668	10476 to 20861	Yes	****	<0.0001
mPFC:H2O vs. LO-OFC:EtOH	17297	12105 to 22490	Yes	****	<0.0001
mPFC:H2O vs. VO-OFC:H2O	16431	11168 to 21694	Yes	****	<0.0001
mPFC:H2O vs. VO-OFC:EtOH	16340	11077 to 21603	Yes	****	<0.0001
mPFC:EtOH vs. LO-OFC:H2O	13736	8543 to 18928	Yes	****	<0.0001
mPFC:EtOH vs. LO-OFC:EtOH	15364	10172 to 20557	Yes	****	<0.0001
mPFC:EtOH vs. VO-OFC:H2O	14498	9235 to 19761	Yes	****	<0.0001
mPFC:EtOH vs. VO-OFC:EtOH	14407	9144 to 19670	Yes	****	<0.0001
LO-OFC:H2O vs. LO-OFC:EtOH	1629	-4304 to 7562	No	ns	0.991
LO-OFC:H2O vs. VO-OFC:H2O	762.8	-5232 to 6758	No	ns	>0.9999
LO-OFC:H2O vs. VO-OFC:EtOH	671.2	-5324 to 6666	No	ns	>0.9999
LO-OFC:EtOH vs. VO-OFC:H2O	-866.1	-6861 to 5129	No	ns	0.9999
LO-OFC:EtOH vs. VO-OFC:EtOH	-957.7	-6952 to 5037	No	ns	0.9997
VO-OFC:H2O vs. VO-OFC:EtOH	-91.6	-6147 to 5964	No	ns	>0.9999

Figure 2 B

Table Analyzed	26DWO Astrocyte Volume				
Two-way ANOVA	Ordinary				
Alpha	0.05				
Source of Variation	% of total variation	P value	P value summary	Significant?	
Interaction	0.08666	0.8121	ns	No	
Subregion	56.74	<0.0001	****	Yes	
Treatment	0.01762	0.6597	ns	No	
ANOVA table	SS (Type III)	DF	MS	F (DFn, DFd)	P value
Interaction	54697533	3	18232511	F (3, 471) = 0.3184	P=0.8121
Subregion	35812955773	3	11937651924	F (3, 471) = 208.4	P<0.0001
Treatment	11119535	1	11119535	F (1, 471) = 0.1942	P=0.6597
Residual	26974067840	471	57269783		
**Tukey's multiple comparisons test**	**Predicted (LS) mean diff.**	**95.00% CI of diff.**	**Below threshold?**	**Summary**	**Adjusted P Value**
ACC:H2O vs. ACC:EtOH	291	-4143 to 4725	No	ns	>0.9999
ACC:H2O vs. mPFC:H2O	-4060	-7965 to -155.4	Yes	*	0.0349
ACC:H2O vs. mPFC:EtOH	-4777	-8976 to -576.9	Yes	*	0.0135
ACC:H2O vs. LO-OFC:H2O	12034	7557 to 16510	Yes	****	<0.0001
ACC:H2O vs. LO-OFC:EtOH	12879	8402 to 17355	Yes	****	<0.0001
ACC:H2O vs. VO-OFC:H2O	16046	11525 to 20568	Yes	****	<0.0001
ACC:H2O vs. VO-OFC:EtOH	16874	12376 to 21373	Yes	****	<0.0001
ACC:EtOH vs. mPFC:H2O	-4351	-8256 to -446.3	Yes	*	0.017
ACC:EtOH vs. mPFC:EtOH	-5068	-9267 to -867.9	Yes	**	0.0064
ACC:EtOH vs. LO-OFC:H2O	11743	7266 to 16219	Yes	****	<0.0001
ACC:EtOH vs. LO-OFC:EtOH	12588	8111 to 17065	Yes	****	<0.0001
ACC:EtOH vs. VO-OFC:H2O	15756	11234 to 20278	Yes	****	<0.0001
ACC:EtOH vs. VO-OFC:EtOH	16583	12085 to 21082	Yes	****	<0.0001
mPFC:H2O vs. mPFC:EtOH	-716.4	-4353 to 2920	No	ns	0.9989
mPFC:H2O vs. LO-OFC:H2O	16094	12141 to 20047	Yes	****	<0.0001
mPFC:H2O vs. LO-OFC:EtOH	16939	12986 to 20892	Yes	****	<0.0001
mPFC:H2O vs. VO-OFC:H2O	20107	16102 to 24111	Yes	****	<0.0001
mPFC:H2O vs. VO-OFC:EtOH	20935	16956 to 24913	Yes	****	<0.0001
mPFC:EtOH vs. LO-OFC:H2O	16810	12566 to 21055	Yes	****	<0.0001
mPFC:EtOH vs. LO-OFC:EtOH	17656	13411 to 21900	Yes	****	<0.0001
mPFC:EtOH vs. VO-OFC:H2O	20823	16531 to 25116	Yes	****	<0.0001
mPFC:EtOH vs. VO-OFC:EtOH	21651	17383 to 25919	Yes	****	<0.0001
LO-OFC:H2O vs. LO-OFC:EtOH	845.3	-3673 to 5364	No	ns	0.9992
LO-OFC:H2O vs. VO-OFC:H2O	4013	-550.7 to 8577	No	ns	0.1322
LO-OFC:H2O vs. VO-OFC:EtOH	4841	300.1 to 9382	Yes	*	0.0273
LO-OFC:EtOH vs. VO-OFC:H2O	3168	-1396 to 7731	No	ns	0.4078
LO-OFC:EtOH vs. VO-OFC:EtOH	3996	-545.2 to 8536	No	ns	0.1316
VO-OFC:H2O vs. VO-OFC:EtOH	827.9	-3758 to 5413	No	ns	0.9994

.

Figure 3A

Table Analyzed	10th PAP-Synaptic Coloc				
Two-way ANOVA	Ordinary				
Alpha	0.05				
Source of Variation	% of total variation	P value	P value summary	Significant?	
Interaction	1.072	0.0159	*	Yes	
Subregion	49.46	<0.0001	****	Yes	
Treatment	0.5236	0.0244	*	Yes	
ANOVA table	SS (Type III)	DF	MS	F (DFn, DFd)	P value
Interaction	0.01229	3	0.004096	F (3, 476) = 3.480	P=0.0159
Subregion	0.5667	3	0.1889	F (3, 476) = 160.5	P<0.0001
Treatment	0.006	1	0.006	F (1, 476) = 5.097	P=0.0244
Residual	0.5602	476	0.001177		
**Tukey's multiple comparisons test**	**Predicted (LS) mean diff.**	**95.00% CI of diff.**	**Below threshold?**	**Summary**	**Adjusted P Value**
ACC:H2O vs. ACC:EtOH	0.01808	-0.002806 to 0.03897	No	ns	0.1459
ACC:H2O vs. mPFC:H2O	0.002078	-0.01620 to 0.02036	No	ns	>0.9999
ACC:H2O vs. mPFC:EtOH	0.01019	-0.008091 to 0.02847	No	ns	0.6892
ACC:H2O vs. LO-OFC:H2O	-0.04176	-0.06265 to -0.02087	Yes	****	<0.0001
ACC:H2O vs. LO-OFC:EtOH	-0.05266	-0.07355 to -0.03177	Yes	****	<0.0001
ACC:H2O vs. VO-OFC:H2O	-0.081	-0.1021 to -0.05989	Yes	****	<0.0001
ACC:H2O vs. VO-OFC:EtOH	-0.06701	-0.08812 to -0.04591	Yes	****	<0.0001
ACC:EtOH vs. mPFC:H2O	-0.01601	-0.03429 to 0.002277	No	ns	0.136
ACC:EtOH vs. mPFC:EtOH	-0.007891	-0.02617 to 0.01039	No	ns	0.8933
ACC:EtOH vs. LO-OFC:H2O	-0.05984	-0.08073 to -0.03895	Yes	****	<0.0001
ACC:EtOH vs. LO-OFC:EtOH	-0.07074	-0.09163 to -0.04985	Yes	****	<0.0001
ACC:EtOH vs. VO-OFC:H2O	-0.09908	-0.1202 to -0.07798	Yes	****	<0.0001
ACC:EtOH vs. VO-OFC:EtOH	-0.08509	-0.1062 to -0.06399	Yes	****	<0.0001
mPFC:H2O vs. mPFC:EtOH	0.008114	-0.007121 to 0.02335	No	ns	0.7372
mPFC:H2O vs. LO-OFC:H2O	-0.04384	-0.06212 to -0.02555	Yes	****	<0.0001
mPFC:H2O vs. LO-OFC:EtOH	-0.05473	-0.07302 to -0.03645	Yes	****	<0.0001
mPFC:H2O vs. VO-OFC:H2O	-0.08308	-0.1016 to -0.06455	Yes	****	<0.0001
mPFC:H2O vs. VO-OFC:EtOH	-0.06909	-0.08762 to -0.05056	Yes	****	<0.0001
mPFC:EtOH vs. LO-OFC:H2O	-0.05195	-0.07023 to -0.03367	Yes	****	<0.0001
mPFC:EtOH vs. LO-OFC:EtOH	-0.06285	-0.08113 to -0.04457	Yes	****	<0.0001
mPFC:EtOH vs. VO-OFC:H2O	-0.09119	-0.1097 to -0.07266	Yes	****	<0.0001
mPFC:EtOH vs. VO-OFC:EtOH	-0.0772	-0.09573 to -0.05867	Yes	****	<0.0001
LO-OFC:H2O vs. LO-OFC:EtOH	-0.0109	-0.03179 to 0.009991	No	ns	0.7572
LO-OFC:H2O vs. VO-OFC:H2O	-0.03924	-0.06035 to -0.01813	Yes	****	<0.0001
LO-OFC:H2O vs. VO-OFC:EtOH	-0.02525	-0.04636 to -0.004148	Yes	**	0.0072
LO-OFC:EtOH vs. VO-OFC:H2O	-0.02834	-0.04945 to -0.007236	Yes	**	0.0013
LO-OFC:EtOH vs. VO-OFC:EtOH	-0.01436	-0.03546 to 0.006750	No	ns	0.4355
VO-OFC:H2O vs. VO-OFC:EtOH	0.01399	-0.007334 to 0.03531	No	ns	0.4848

.

Figure 3B

**Table Analyzed**	**26DWO PAP-Synaptic Coloc**				
Two-way ANOVA	Ordinary				
Alpha	0.05				
Source of Variation	% of total variation	P value	P value summary	Significant?	
Interaction	5.654	<0.0001	****	Yes	
Subregion	54.37	<0.0001	****	Yes	
Treatment	2.7	<0.0001	****	Yes	
ANOVA table	SS (Type III)	DF	MS	F (DFn, DFd)	P value
Interaction	0.07767	3	0.02589	F (3, 502) = 24.70	P<0.0001
Subregion	0.747	3	0.249	F (3, 502) = 237.6	P<0.0001
Treatment	0.03709	1	0.03709	F (1, 502) = 35.38	P<0.0001
Residual	0.5262	502	0.001048		
**Tukey's multiple comparisons test**	**Predicted (LS) mean diff.**	**95.00% CI of diff.**	**Below threshold?**	**Summary**	**Adjusted P Value**
ACC:H2O vs. ACC:EtOH	0.02283	0.003870 to 0.04180	Yes	**	0.0066
ACC:H2O vs. mPFC:H2O	0.03108	0.01438 to 0.04778	Yes	****	<0.0001
ACC:H2O vs. mPFC:EtOH	0.02574	0.009042 to 0.04244	Yes	****	<0.0001
ACC:H2O vs. LO-OFC:H2O	-0.04216	-0.06130 to -0.02301	Yes	****	<0.0001
ACC:H2O vs. LO-OFC:EtOH	-0.0465	-0.06564 to -0.02735	Yes	****	<0.0001
ACC:H2O vs. VO-OFC:H2O	-0.09188	-0.1111 to -0.07264	Yes	****	<0.0001
ACC:H2O vs. VO-OFC:EtOH	-0.03425	-0.05349 to -0.01501	Yes	****	<0.0001
ACC:EtOH vs. mPFC:H2O	0.008242	-0.008458 to 0.02494	No	ns	0.8062
ACC:EtOH vs. mPFC:EtOH	0.002908	-0.01379 to 0.01961	No	ns	0.9995
ACC:EtOH vs. LO-OFC:H2O	-0.06499	-0.08414 to -0.04584	Yes	****	<0.0001
ACC:EtOH vs. LO-OFC:EtOH	-0.06933	-0.08848 to -0.05018	Yes	****	<0.0001
ACC:EtOH vs. VO-OFC:H2O	-0.1147	-0.1340 to -0.09547	Yes	****	<0.0001
ACC:EtOH vs. VO-OFC:EtOH	-0.05708	-0.07632 to -0.03784	Yes	****	<0.0001
mPFC:H2O vs. mPFC:EtOH	-0.005334	-0.01941 to 0.008743	No	ns	0.9444
mPFC:H2O vs. LO-OFC:H2O	-0.07323	-0.09014 to -0.05633	Yes	****	<0.0001
mPFC:H2O vs. LO-OFC:EtOH	-0.07757	-0.09448 to -0.06067	Yes	****	<0.0001
mPFC:H2O vs. VO-OFC:H2O	-0.123	-0.1400 to -0.1059	Yes	****	<0.0001
mPFC:H2O vs. VO-OFC:EtOH	-0.06532	-0.08234 to -0.04831	Yes	****	<0.0001
mPFC:EtOH vs. LO-OFC:H2O	-0.0679	-0.08480 to -0.05099	Yes	****	<0.0001
mPFC:EtOH vs. LO-OFC:EtOH	-0.07224	-0.08915 to -0.05533	Yes	****	<0.0001
mPFC:EtOH vs. VO-OFC:H2O	-0.1176	-0.1346 to -0.1006	Yes	****	<0.0001
mPFC:EtOH vs. VO-OFC:EtOH	-0.05999	-0.07700 to -0.04298	Yes	****	<0.0001
LO-OFC:H2O vs. LO-OFC:EtOH	-0.00434	-0.02367 to 0.01499	No	ns	0.9974
LO-OFC:H2O vs. VO-OFC:H2O	-0.04972	-0.06914 to -0.03030	Yes	****	<0.0001
LO-OFC:H2O vs. VO-OFC:EtOH	0.007908	-0.01151 to 0.02733	No	ns	0.9198
LO-OFC:EtOH vs. VO-OFC:H2O	-0.04538	-0.06480 to -0.02596	Yes	****	<0.0001
LO-OFC:EtOH vs. VO-OFC:EtOH	0.01225	-0.007172 to 0.03167	No	ns	0.5379
VO-OFC:H2O vs. VO-OFC:EtOH	0.05763	0.03812 to 0.07714	Yes	****	<0.0001

Accompanies Figure 4.

**Table Analyzed**	**Spine Density**				
Two-way ANOVA	Ordinary				
Alpha	0.05				
Source of Variation	% of total variation	P value	P value summary	Significant?	
Interaction	0.9223	0.337	ns	No	
Treatment	3.073	0.011	*	Yes	
Subregion	0.2171	0.3723	ns	No	
ANOVA table	SS	DF	MS	F (DFn, DFd)	P value
Interaction	0.08207	3	0.02736	F (3, 352) = 1.130	P=0.3370
Treatment	0.2735	3	0.09115	F (3, 352) = 3.764	P=0.0110
Subregion	0.01932	1	0.01932	F (1, 352) = 0.7979	P=0.3723
Residual	8.524	352	0.02422		
Difference between column means					
Mean of H_2O	0.9292				
Mean of EtOH	0.9145				
Difference between means	0.01465				
SE of difference	0.0164				
95% CI of difference	-0.01761 to 0.04691				
Normality of Residuals					
Test name	Statistics	P value	Passed normality test (alpha=0.05)?	P value summary	
D'Agostino-Pearson omnibus (K2)	16.32	0.0003	No	***	
Anderson-Darling (A2*)	1.431	0.0011	No	**	
Shapiro-Wilk (W)	0.9776	<0.0001	No	****	
Kolmogorov-Smirnov (distance)	0.05498	0.0108	No	*	
Data summary					
Number of columns (Subregion)	2				
Number of rows (Treatment)	4				
Number of values	360				
Compare cell means regardless of rows and columns					
Number of families	1				
Number of comparisons per family	28				
Alpha	0.05				
**Tukey's multiple comparisons test**	Mean Diff.	95.00% CI of diff.	Below threshold?	Summary	Adjusted P Value
ACC:H_2O vs. ACC:EtOH	0.06107	-0.03897 to 0.1611	No	ns	0.5784
ACC:H_2O vs. mPFC:H_2O	0.09903	-0.001011 to 0.1991	No	ns	0.0546
ACC:H_2O vs. mPFC:EtOH	0.07684	-0.02320 to 0.1769	No	ns	0.2739
ACC:H_2O vs. LO-OFC:H_2O	0.01307	-0.08697 to 0.1131	No	ns	>0.9999
ACC:H_2O vs. LO-OFC:EtOH	0.02964	-0.07039 to 0.1297	No	ns	0.9856
ACC:H_2O vs. VO-OFC:H_2O	0.06911	-0.03093 to 0.1691	No	ns	0.4127
ACC:H_2O vs. VO-OFC:EtOH	0.07227	-0.02777 to 0.1723	No	ns	0.3526
ACC:EtOH vs. mPFC:H_2O	0.03796	-0.06208 to 0.1380	No	ns	0.9433
ACC:EtOH vs. mPFC:EtOH	0.01577	-0.08427 to 0.1158	No	ns	0.9997
ACC:EtOH vs. LO-OFC:H_2O	-0.048	-0.1480 to 0.05204	No	ns	0.8265
ACC:EtOH vs. LO-OFC:EtOH	-0.03143	-0.1315 to 0.06861	No	ns	0.9798
ACC:EtOH vs. VO-OFC:H_2O	0.008041	-0.09200 to 0.1081	No	ns	>0.9999
ACC:EtOH vs. VO-OFC:EtOH	0.0112	-0.08884 to 0.1112	No	ns	>0.9999
mPFC:H_2O vs. mPFC:EtOH	-0.02219	-0.1222 to 0.07785	No	ns	0.9976
mPFC:H_2O vs. LO-OFC:H_2O	-0.08596	-0.1860 to 0.01408	No	ns	0.1522
mPFC:H_2O vs. LO-OFC:EtOH	-0.06938	-0.1694 to 0.03065	No	ns	0.4073
mPFC:H_2O vs. VO-OFC:H_2O	-0.02992	-0.1300 to 0.07012	No	ns	0.9848
mPFC:H_2O vs. VO-OFC:EtOH	-0.02676	-0.1268 to 0.07328	No	ns	0.9922
mPFC:EtOH vs. LO-OFC:H_2O	-0.06377	-0.1638 to 0.03627	No	ns	0.5218
mPFC:EtOH vs. LO-OFC:EtOH	-0.04719	-0.1472 to 0.05284	No	ns	0.8387
mPFC:EtOH vs. VO-OFC:H_2O	-0.007726	-0.1078 to 0.09231	No	ns	>0.9999
mPFC:EtOH vs. VO-OFC:EtOH	-0.004571	-0.1046 to 0.09547	No	ns	>0.9999
LO-OFC:H_2O vs. LO-OFC:EtOH	0.01657	-0.08346 to 0.1166	No	ns	0.9996
LO-OFC:H_2O vs. VO-OFC:H_2O	0.05604	-0.04400 to 0.1561	No	ns	0.682
LO-OFC:H_2O vs. VO-OFC:EtOH	0.0592	-0.04084 to 0.1592	No	ns	0.6175
LO-OFC:EtOH vs. VO-OFC:H_2O	0.03947	-0.06057 to 0.1395	No	ns	0.9307
LO-OFC:EtOH vs. VO-OFC:EtOH	0.04262	-0.05742 to 0.1427	No	ns	0.8988
VO-OFC:H_2O vs. VO-OFC:EtOH	0.003155	-0.09688 to 0.1032	No	ns	>0.9999

.

Figure 4D

Table Analyzed	26DWO Immature Dendritic Spines				
Two-way ANOVA	Ordinary				
Alpha	0.05				
Source of Variation	% of total variation	P value	P value summary	Significant?	
Interaction	3.32	0.3192	ns	No	
Subregion	3.72	0.2696	ns	No	
Treatment	3.137	0.0699	ns	No	
ANOVA table	SS (Type III)	DF	MS	F (DFn, DFd)	P value
Interaction	740.4	3	246.8	F (3, 86) = 1.188	P=0.3192
Subregion	829.7	3	276.6	F (3, 86) = 1.331	P=0.2696
Treatment	699.8	1	699.8	F (1, 86) = 3.368	P=0.0699
Residual	17868	86	207.8		
**Tukey's multiple comparisons tes**	**Predicted (LS) mean diff.**	**95.00% CI of diff.**	**Below threshold**	**Summary**	**Adjusted P Value**
ACC:H2O vs. ACC:EtOH	6	-26.70 to 38.70	No	ns	0.9991
ACC:H2O vs. mPFC:H2O	-12.52	-34.01 to 8.976	No	ns	0.6156
ACC:H2O vs. mPFC:EtOH	2.857	-20.47 to 26.19	No	ns	>0.9999
ACC:H2O vs. LO-OFC:H2O	0.625	-24.90 to 26.15	No	ns	>0.9999
ACC:H2O vs. LO-OFC:EtOH	3.333	-23.78 to 30.45	No	ns	>0.9999
ACC:H2O vs. VO-OFC:H2O	-1.5	-25.34 to 22.34	No	ns	>0.9999
ACC:H2O vs. VO-OFC:EtOH	2	-21.57 to 25.57	No	ns	>0.9999
ACC:EtOH vs. mPFC:H2O	-18.52	-45.52 to 8.490	No	ns	0.4044
ACC:EtOH vs. mPFC:EtOH	-3.143	-31.63 to 25.35	No	ns	>0.9999
ACC:EtOH vs. LO-OFC:H2O	-5.375	-35.69 to 24.94	No	ns	0.9993
ACC:EtOH vs. LO-OFC:EtOH	-2.667	-34.33 to 29.00	No	ns	>0.9999
ACC:EtOH vs. VO-OFC:H2O	-7.5	-36.41 to 21.41	No	ns	0.9924
ACC:EtOH vs. VO-OFC:EtOH	-4	-32.68 to 24.68	No	ns	0.9999
mPFC:H2O vs. mPFC:EtOH	15.37	1.089 to 29.66	Yes	*	0.0258
mPFC:H2O vs. LO-OFC:H2O	13.14	-4.508 to 30.79	No	ns	0.2986
mPFC:H2O vs. LO-OFC:EtOH	15.85	-4.027 to 35.72	No	ns	0.2192
mPFC:H2O vs. VO-OFC:H2O	11.02	-4.081 to 26.11	No	ns	0.3238
mPFC:H2O vs. VO-OFC:EtOH	14.52	-0.1490 to 29.18	No	ns	0.0544
mPFC:EtOH vs. LO-OFC:H2O	-2.232	-22.08 to 17.62	No	ns	>0.9999
mPFC:EtOH vs. LO-OFC:EtOH	0.4762	-21.38 to 22.33	No	ns	>0.9999
mPFC:EtOH vs. VO-OFC:H2O	-4.357	-21.97 to 13.26	No	ns	0.9943
mPFC:EtOH vs. VO-OFC:EtOH	-0.8571	-18.11 to 16.39	No	ns	>0.9999
LO-OFC:H2O vs. LO-OFC:EtOH	2.708	-21.48 to 26.89	No	ns	>0.9999
LO-OFC:H2O vs. VO-OFC:H2O	-2.125	-22.57 to 18.32	No	ns	>0.9999
LO-OFC:H2O vs. VO-OFC:EtOH	1.375	-18.75 to 21.50	No	ns	>0.9999
LO-OFC:EtOH vs. VO-OFC:H2O	-4.833	-27.22 to 17.56	No	ns	0.9976
LO-OFC:EtOH vs. VO-OFC:EtOH	-1.333	-23.44 to 20.77	No	ns	>0.9999
VO-OFC:H2O vs. VO-OFC:EtOH	3.5	-14.43 to 21.43	No	ns	0.9987

Figure 4E.

**Table Analyzed**	**26DWO Intermediate Dendritic Spines**				
Two-way ANOVA	Ordinary				
Alpha	0.05				
Source of Variation	% of total variation	P value	P value summary	Significant?	
Interaction	5.213	<0.0001	****	Yes	
Subregion	7.107	<0.0001	****	Yes	
Treatment	0.7949	0.045	*	Yes	
ANOVA table	SS (Type III)	DF	MS	F (DFn, DFd)	P value
Interaction	10387	3	3462	F (3, 440) = 8.833	P<0.0001
Subregion	14159	3	4720	F (3, 440) = 12.04	P<0.0001
Treatment	1584	1	1584	F (1, 440) = 4.040	P=0.0450
Residual	172475	440	392		
**Tukey's multiple comparisons test**	**Predicted (LS) mean diff.**	**95.00% CI of diff.**	**Below threshold?**	**Summary**	**Adjusted P Value**
ACC:H20 vs. ACC:EtOH	-7.978	-20.69 to 4.734	No	ns	0.5438
ACC:H20 vs. mPFC:H20	-1.014	-12.06 to 10.04	No	ns	>0.9999
ACC:H20 vs. mPFC:EtOH	-7.856	-18.86 to 3.153	No	ns	0.3701
ACC:H20 vs. LO-OFC:H20	-25.53	-38.25 to -12.82	Yes	****	<0.0001
ACC:H20 vs. LO-OFC:EtOH	-11.73	-24.45 to 0.9787	No	ns	0.0948
ACC:H20 vs. VO-OFC:H20	-2.511	-15.22 to 10.20	No	ns	0.9989
ACC:H20 vs. VO-OFC:EtOH	-17.2	-29.91 to -4.488	Yes	**	0.0012
ACC:EtOH vs. mPFC:H20	6.964	-4.087 to 18.01	No	ns	0.5383
ACC:EtOH vs. mPFC:EtOH	0.1222	-10.89 to 11.13	No	ns	>0.9999
ACC:EtOH vs. LO-OFC:H20	-17.56	-30.27 to -4.844	Yes	***	0.0008
ACC:EtOH vs. LO-OFC:EtOH	-3.756	-16.47 to 8.956	No	ns	0.986
ACC:EtOH vs. VO-OFC:H20	5.467	-7.245 to 18.18	No	ns	0.895
ACC:EtOH vs. VO-OFC:EtOH	-9.222	-21.93 to 3.490	No	ns	0.348
mPFC:H20 vs. mPFC:EtOH	-6.842	-15.88 to 2.198	No	ns	0.2931
mPFC:H20 vs. LO-OFC:H20	-24.52	-35.57 to -13.47	Yes	****	<0.0001
mPFC:H20 vs. LO-OFC:EtOH	-10.72	-21.77 to 0.3311	No	ns	0.0647
mPFC:H20 vs. VO-OFC:H20	-1.497	-12.55 to 9.553	No	ns	>0.9999
mPFC:H20 vs. VO-OFC:EtOH	-16.19	-27.24 to -5.136	Yes	***	0.0003
mPFC:EtOH vs. LO-OFC:H20	-17.68	-28.69 to -6.669	Yes	****	<0.0001
mPFC:EtOH vs. LO-OFC:EtOH	-3.878	-14.89 to 7.131	No	ns	0.9621
mPFC:EtOH vs. VO-OFC:H20	5.344	-5.664 to 16.35	No	ns	0.8187
mPFC:EtOH vs. VO-OFC:EtOH	-9.344	-20.35 to 1.664	No	ns	0.1641
LO-OFC:H20 vs. LO-OFC:EtOH	13.8	1.088 to 26.51	Yes	*	0.0226
LO-OFC:H20 vs. VO-OFC:H20	23.02	10.31 to 35.73	Yes	****	<0.0001
LO-OFC:H20 vs. VO-OFC:EtOH	8.333	-4.379 to 21.05	No	ns	0.4854
LO-OFC:EtOH vs. VO-OFC:H20	9.222	-3.490 to 21.93	No	ns	0.348
LO-OFC:EtOH vs. VO-OFC:EtOH	-5.467	-18.18 to 7.245	No	ns	0.895
VO-OFC:H20 vs. VO-OFC:EtOH	-14.69	-27.40 to -1.977	Yes	*	0.0112

Figure 4F

**Table Analyzed**	**26DWO Mature Dendritic Spines**				
Two-way ANOVA	Ordinary				
Alpha	0.05				
Source of Variation	% of total variation	P value	P value summary	Significant?	
Interaction	5.413	<0.0001	****	Yes	
Subregion	7.622	<0.0001	****	Yes	
Treatment	0.3433	0.2168	ns	No	
ANOVA table	SS (Type III)	DF	MS	F (DFn, DFd)	P value
Interaction	5619	3	1873	F (3, 390) = 8.043	P<0.0001
Subregion	7912	3	2637	F (3, 390) = 11.32	P<0.0001
Treatment	356.4	1	356.4	F (1, 390) = 1.530	P=0.2168
Residual	90826	390	232.9		
**Tukey's multiple comparisons test**	**Predicted (LS) mean diff.**	**95.00% CI of diff.**	**Below threshold?**	**Summary**	**Adjusted P Value**
ACC:H2O vs. ACC:EtOH	5.946	-4.205 to 16.10	No	ns	0.6307
ACC:H2O vs. mPFC:H2O	4.595	-4.180 to 13.37	No	ns	0.7527
ACC:H2O vs. mPFC:EtOH	5.798	-2.959 to 14.55	No	ns	0.4711
ACC:H2O vs. LO-OFC:H2O	21.24	10.06 to 32.41	Yes	****	<0.0001
ACC:H2O vs. LO-OFC:EtOH	9.737	-0.3525 to 19.83	No	ns	0.0674
ACC:H2O vs. VO-OFC:H2O	4.785	-5.305 to 14.87	No	ns	0.8355
ACC:H2O vs. VO-OFC:EtOH	17.16	6.803 to 27.51	Yes	****	<0.0001
ACC:EtOH vs. mPFC:H2O	-1.351	-10.26 to 7.563	No	ns	0.9998
ACC:EtOH vs. mPFC:EtOH	-0.1481	-9.044 to 8.747	No	ns	>0.9999
ACC:EtOH vs. LO-OFC:H2O	15.29	4.007 to 26.58	Yes	**	0.0011
ACC:EtOH vs. LO-OFC:EtOH	3.791	-6.419 to 14.00	No	ns	0.9496
ACC:EtOH vs. VO-OFC:H2O	-1.161	-11.37 to 9.049	No	ns	>0.9999
ACC:EtOH vs. VO-OFC:EtOH	11.21	0.7396 to 21.68	Yes	*	0.0262
mPFC:H2O vs. mPFC:EtOH	1.203	-6.083 to 8.488	No	ns	0.9996
mPFC:H2O vs. LO-OFC:H2O	16.64	6.578 to 26.71	Yes	****	<0.0001
mPFC:H2O vs. LO-OFC:EtOH	5.142	-3.701 to 13.98	No	ns	0.6395
mPFC:H2O vs. VO-OFC:H2O	0.1896	-8.653 to 9.033	No	ns	>0.9999
mPFC:H2O vs. VO-OFC:EtOH	12.56	3.419 to 21.71	Yes	***	0.0009
mPFC:EtOH vs. LO-OFC:H2O	15.44	5.391 to 25.49	Yes	***	0.0001
mPFC:EtOH vs. LO-OFC:EtOH	3.939	-4.886 to 12.76	No	ns	0.8745
mPFC:EtOH vs. VO-OFC:H2O	-1.013	-9.838 to 7.811	No	ns	>0.9999
mPFC:EtOH vs. VO-OFC:EtOH	11.36	2.234 to 20.49	Yes	**	0.0042
LO-OFC:H2O vs. LO-OFC:EtOH	-11.5	-22.73 to -0.2716	Yes	*	0.0403
LO-OFC:H2O vs. VO-OFC:H2O	-16.45	-27.68 to -5.224	Yes	***	0.0003
LO-OFC:H2O vs. VO-OFC:EtOH	-4.079	-15.55 to 7.388	No	ns	0.9599
LO-OFC:EtOH vs. VO-OFC:H2O	-4.952	-15.10 to 5.196	No	ns	0.8141
LO-OFC:EtOH vs. VO-OFC:EtOH	7.421	-2.991 to 17.83	No	ns	0.3711
VO-OFC:H2O vs. VO-OFC:EtOH	12.37	1.961 to 22.79	Yes	**	0.0079

.

Figure 5B

**Table Analyzed**	**26DWO NLG1-NRXN**				
Two-way ANOVA	Ordinary				
Alpha	0.05				
Source of Variation	% of total variation	P value	P value summary	Significant?	
Interaction	4.626	0.0001	***	Yes	
Subregion	31.12	<0.0001	****	Yes	
Treatment	3.041	0.0002	***	Yes	
ANOVA table	SS (Type III)	DF	MS	F (DFn, DFd)	P value
Interaction	1704	3	568	F (3, 286) = 7.137	P=0.0001
Subregion	11464	3	3821	F (3, 286) = 48.01	P<0.0001
Treatment	1120	1	1120	F (1, 286) = 14.08	P=0.0002
Residual	22762	286	79.59		
**Tukey's multiple comparisons test**	**Predicted (LS) mean diff.**	**95.00% CI of diff.**	**Below threshold?**	**Summary**	**Adjusted P Value**
ACC:H_2O vs. ACC:EtOH	10.6	3.566 to 17.63	Yes	***	0.0002
ACC:H_2O vs. mPFC:H_2O	15.36	9.213 to 21.50	Yes	****	<0.0001
ACC:H_2O vs. mPFC:EtOH	16.46	10.32 to 22.61	Yes	****	<0.0001
ACC:H_2O vs. LO-OFC:H_2O	9.667	2.633 to 16.70	Yes	***	0.0009
ACC:H_2O vs. LO-OFC:EtOH	6.967	-0.06694 to 14.00	No	ns	0.0543
ACC:H_2O vs. VO-OFC:H_2O	19.16	12.13 to 26.19	Yes	****	<0.0001
ACC:H_2O vs. VO-OFC:EtOH	26.38	19.35 to 33.42	Yes	****	<0.0001
ACC:EtOH vs. mPFC:H_2O	4.758	-1.387 to 10.90	No	ns	0.2631
ACC:EtOH vs. mPFC:EtOH	5.863	-0.2813 to 12.01	No	ns	0.0736
ACC:EtOH vs. LO-OFC:H_2O	-0.9333	-7.967 to 6.100	No	ns	>0.9999
ACC:EtOH vs. LO-OFC:EtOH	-3.633	-10.67 to 3.400	No	ns	0.7635
ACC:EtOH vs. VO-OFC:H_2O	8.56	1.526 to 15.59	Yes	**	0.0059
ACC:EtOH vs. VO-OFC:EtOH	15.78	8.750 to 22.82	Yes	****	<0.0001
mPFC:H_2O vs. mPFC:EtOH	1.105	-3.997 to 6.208	No	ns	0.9979
mPFC:H_2O vs. LO-OFC:H_2O	-5.691	-11.84 to 0.4533	No	ns	0.0921
mPFC:H_2O vs. LO-OFC:EtOH	-8.391	-14.54 to -2.247	Yes	**	0.001
mPFC:H_2O vs. VO-OFC:H_2O	3.802	-2.342 to 9.947	No	ns	0.5593
mPFC:H_2O vs. VO-OFC:EtOH	11.03	4.881 to 17.17	Yes	****	<0.0001
mPFC:EtOH vs. LO-OFC:H_2O	-6.796	-12.94 to -0.6520	Yes	*	0.0187
mPFC:EtOH vs. LO-OFC:EtOH	-9.496	-15.64 to -3.352	Yes	****	<0.0001
mPFC:EtOH vs. VO-OFC:H_2O	2.697	-3.448 to 8.841	No	ns	0.8827
mPFC:EtOH vs. VO-OFC:EtOH	9.92	3.776 to 16.06	Yes	****	<0.0001
LO-OFC:H_2O vs. LO-OFC:EtOH	-2.7	-9.734 to 4.334	No	ns	0.9392
LO-OFC:H_2O vs. VO-OFC:H_2O	9.493	2.460 to 16.53	Yes	**	0.0013
LO-OFC:H_2O vs. VO-OFC:EtOH	16.72	9.683 to 23.75	Yes	****	<0.0001
LO-OFC:EtOH vs. VO-OFC:H_2O	12.19	5.160 to 19.23	Yes	****	<0.0001
LO-OFC:EtOH vs. VO-OFC:EtOH	19.42	12.38 to 26.45	Yes	****	<0.0001
VO-OFC:H_2O vs. VO-OFC:EtOH	7.223	0.1897 to 14.26	Yes	*	0.0394

.

Table Analyzed	26DWO NLG3-NRXN				
Two-way ANOVA	Ordinary				
Alpha	0.05				
Source of Variation	% of total variation	P value	P value summary	Significant?	
Interaction	6.356	<0.0001	****	Yes	
Subregion	16.12	<0.0001	****	Yes	
Treatment	1.793	0.0103	*	Yes	
ANOVA table	SS (Type III)	DF	MS	F (DFn, DFd)	P value
Interaction	2510	3	836.6	F (3, 286) = 7.874	P<0.0001
Subregion	6364	3	2121	F (3, 286) = 19.97	P<0.0001
Treatment	708	1	708	F (1, 286) = 6.664	P=0.0103
Residual	30387	286	106.2		
**Tukey's multiple comparisons test**	**Predicted (LS) mean diff.**	**95.00% CI of diff.**	**Below threshold?**	**Summary**	**Adjusted P Value**
ACC:H_2O vs. ACC:EtOH	9	0.8732 to 17.13	Yes	*	0.0184
ACC:H_2O vs. mPFC:H_2O	11.6	4.562 to 18.64	Yes	****	<0.0001
ACC:H_2O vs. mPFC:EtOH	7.2	0.1620 to 14.24	Yes	*	0.0408
ACC:H_2O vs. LO-OFC:H_2O	5.178	-3.172 to 13.53	No	ns	0.5565
ACC:H_2O vs. LO-OFC:EtOH	5.733	-2.616 to 14.08	No	ns	0.4195
ACC:H_2O vs. VO-OFC:H_2O	13.7	5.573 to 21.83	Yes	****	<0.0001
ACC:H_2O vs. VO-OFC:EtOH	21.6	13.47 to 29.73	Yes	****	<0.0001
ACC:EtOH vs. mPFC:H_2O	2.6	-4.438 to 9.638	No	ns	0.9502
ACC:EtOH vs. mPFC:EtOH	-1.8	-8.838 to 5.238	No	ns	0.994
ACC:EtOH vs. LO-OFC:H_2O	-3.822	-12.17 to 4.527	No	ns	0.8578
ACC:EtOH vs. LO-OFC:EtOH	-3.267	-11.62 to 5.083	No	ns	0.933
ACC:EtOH vs. VO-OFC:H_2O	4.7	-3.427 to 12.83	No	ns	0.6436
ACC:EtOH vs. VO-OFC:EtOH	12.6	4.473 to 20.73	Yes	****	<0.0001
mPFC:H_2O vs. mPFC:EtOH	-4.4	-10.15 to 1.347	No	ns	0.2768
mPFC:H_2O vs. LO-OFC:H_2O	-6.422	-13.72 to 0.8718	No	ns	0.1303
mPFC:H_2O vs. LO-OFC:EtOH	-5.867	-13.16 to 1.427	No	ns	0.219
mPFC:H_2O vs. VO-OFC:H_2O	2.1	-4.938 to 9.138	No	ns	0.9848
mPFC:H_2O vs. VO-OFC:EtOH	10	2.962 to 17.04	Yes	***	0.0005
mPFC:EtOH vs. LO-OFC:H_2O	-2.022	-9.316 to 5.272	No	ns	0.9902
mPFC:EtOH vs. LO-OFC:EtOH	-1.467	-8.761 to 5.827	No	ns	0.9987
mPFC:EtOH vs. VO-OFC:H_2O	6.5	-0.5380 to 13.54	No	ns	0.0941
mPFC:EtOH vs. VO-OFC:EtOH	14.4	7.362 to 21.44	Yes	****	<0.0001
LO-OFC:H_2O vs. LO-OFC:EtOH	0.5556	-8.011 to 9.122	No	ns	>0.9999
LO-OFC:H_2O vs. VO-OFC:H_2O	8.522	0.1727 to 16.87	Yes	*	0.0417
LO-OFC:H_2O vs. VO-OFC:EtOH	16.42	8.073 to 24.77	Yes	****	<0.0001
LO-OFC:EtOH vs. VO-OFC:H_2O	7.967	-0.3828 to 16.32	No	ns	0.0737
LO-OFC:EtOH vs. VO-OFC:EtOH	15.87	7.517 to 24.22	Yes	****	<0.0001
VO-OFC:H_2O vs. VO-OFC:EtOH	7.9	-0.2268 to 16.03	No	ns	0.0635

Figure S1: All Statistical Analyses Conducted for the Study

PND 46 Astrocyte Volume (Figure 2B)

	H2O				EtOH				
	ACC	mPFC	LO-OFC	VO-OFC		ACC	mPFC	LO-OFC	VO-OFC
Mean	21241.11	25875.93	10207.51	9444.724	Mean	20590.53	23943.1	8578.654	9536.325
SEM	±1574.199	±1320.006	±948.9464	±760.1075	SEM	±1577.566	±1218.267	±455.4112	±597.1823

PND 72 Astrocyte Volume (Figure 2D)

	H2O				EtOH				
	ACC	mPFC	LO-OFC	VO-OFC		ACC	mPFC	LO-OFC	VO-OFC
Mean	22583.12	26643.34	10549.57	7171.821	Mean	22292.15	27359.79	9704.246	5708.709
SEM	±1573.77	±853.4966	±954.58	±727.9249	SEM	±1256.7	±1023.595	±532.8143	±262.4512

PND 46 Astrocyte-Synaptic Interactions (Figure 3B)

	H2O				EtOH				
	ACC	mPFC	LO-OFC	VO-OFC		ACC	mPFC	LO-OFC	VO-OFC
Mean	0.051068	0.04899	0.092826	0.132066	Mean	0.032985	0.040877	0.103724	0.11808
SEM	±0.003792	±0.003361	±0.006336	±0.007241	SEM	±0.001791	±0.00278	±0.004435	±0.006173

PND 72 Astrocyte-Synaptic Interactions (Figure 3D)

	H2O				EtOH				
	ACC	mPFC	LO-OFC	VO-OFC		ACC	mPFC	LO-OFC	VO-OFC
Mean	0.057309	0.026233	0.099465	0.149188	Mean	0.034475	0.031567	0.103806	0.091557
SEM	±0.003903	±0.001636	±0.006104	±0.00822	SEM	±0.001832	±0.00157	±0.005749	±0.00506

PND 72 Immature Spines (Figure 4D)

	H2O				EtOH				
	ACC	mPFC	LO-OFC	VO-OFC		ACC	mPFC	LO-OFC	VO-OFC
Mean	15	27.51515	14.375	16.5	Mean	9	12.14286	11.66667	13
SEM	±3.271085	±3.966985	±1.889232	±1.671961	SEM	±0.57735	±0.64499	±2.108185	±1.281025

PND 72 Intermediate Spines (Figure 4D)

	H2O				EtOH				
	ACC	mPFC	LO-OFC	VO-OFC		ACC	mPFC	LO-OFC	VO-OFC
Mean	60.11111	61.125	85.64444	62.62222	Mean	68.08889	67.96667	71.84444	77.31111
SEM	±3.054591	±2.653678	±1.756993	±3.137632	SEM	±2.911805	±2.025459	±2.385553	±2.617506

PND 72 Mature Spines (Figure 4D)

	H2O				EtOH				
	ACC	mPFC	LO-OFC	VO-OFC		ACC	mPFC	LO-OFC	VO-OFC
Mean	38.23688	33.64198	17	33.45238	Mean	32.2909	32.43902	28.5	21.07895

**SEM**	±2.579452	±1.954601	±1.308366	±2.566921	**SEM**	±2.193903	±1.785307	±2.217094	±1.641557
**PND 72 Neuroligin 1 – Neurexin Colocalization (Figure 5B)**									
**H2O**					**EtOH**				
	**ACC**	**mPFC**	**LO-OFC**	**VO-OFC**		**ACC**	**mPFC**	**LO-OFC**	**VO-OFC**
**Mean**	38.2	22.84211	28.53333	19.04	**Mean**	27.6	21.73684	31.23333	11.81667
**SEM**	±1.962522	±1.399022	±1.38193	±1.243554	**SEM**	±1.38763	±1.347402	±1.5497	±0.828544
**PND 72 Neuroligin 3 – Neurexin Colocalization (Figure 6B)**									
**H2O**					**EtOH**				
	**ACC**	**mPFC**	**LO-OFC**	**VO-OFC**		**ACC**	**mPFC**	**LO-OFC**	**VO-OFC**
**Mean**	34.4	22.8	29.22222	20.7	**Mean**	25.4	27.2	28.66667	12.8
**SEM**	±1.701656	±1.181735	±2.010892	±1.194961	**SEM**	±1.337006	±2.001101	±1.687055	±1.13198

Figure S2: Mean ± SEM for all data sets.

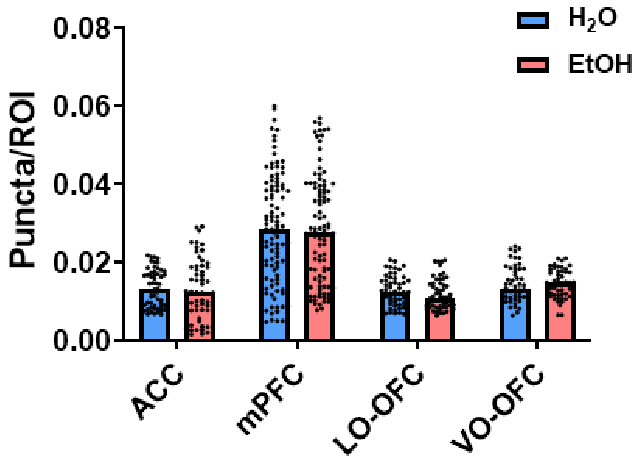

Figure S3. Data demonstrating no effect of treatment on PSD-95 expression in any PFC subregions at PND72 (F (1, 502) = 0.05115, p = 0.8212), when significant AIE-induced loss of PAP-synaptic interactions occur. It should be noted that there is a subregion effect (F (3, 502) = 101.9, p < 0.0001), *post hoc* analysis reveals that this is due to the presence of significantly more PSD-95 puncta within the mPFC when compared to all other subregions (p < 0.0001, for all comparisons). Data presented as mean ± SEM along with individual data points. Analysis: two-way ANOVA with Tukey's post-hoc comparison, n=5/treatment group.

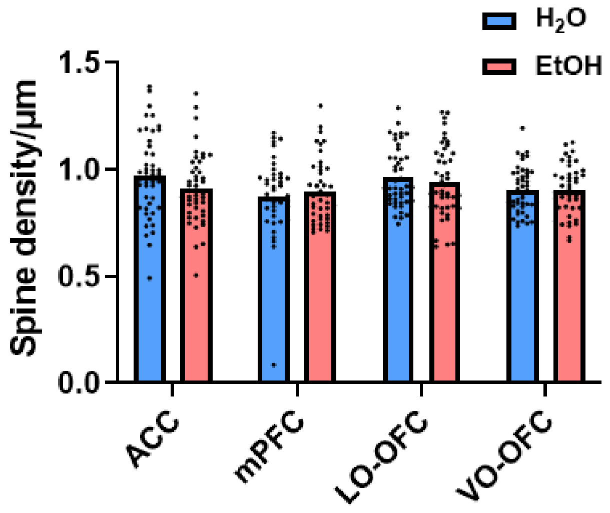

Figure S4. Data demonstrating that there was no significant effect of subregion on spine density (F (1, 352) = 0.7979, p = 0.3723). There are no treatment effects when comparing AIE to control within subregions (p > 0.05). Average spine densities were calculated using 2-3 separate dendrites from at least 4-5 separate image stacks per animal for a total of 15 dendritic branches/animal, n=3/treatment group). Data presented as mean ± SEM along with individual data points. Analysis: two-way ANOVA with Tukey's post-hoc comparison.

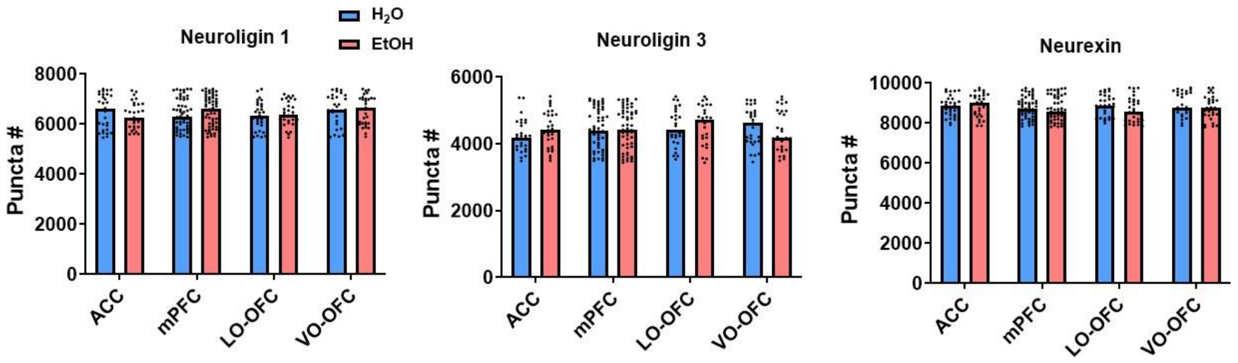

Figure S5. Data demonstrating that there is no significant AIE-induced change in the expression of neuroligin 1 ($F (1, 278) = 0.009163$, $p = 0.9238$), neuroligin 3 ($F (1, 278) = 0.2155$, $p = 0.6429$) or their presynaptic partner, neurexin ($F (1, 272) = 1.710$, $p = 0.1921$), indicating that a loss of these proteins is not a driver of AIE-induced loss PAP-synaptic proximity. Data presented as mean ± SEM along with individual data points. Three 5 μm image stacks from randomly selected hemispheres were captured from 3 separate brain slices per animal (n=9-10 brains/treatment group). Analysis: two-way ANOVA with Tukey's post-hoc comparison.
